# Notes and News

**Published:** 1904-07-09

**Authors:** 


### Notes and News.

On Wednesday next Princess Louise will open the new buildings of the North-Eastern Hospital for Children at 3.15.

A petition in favour of the compulsory teaching of hygiene and temperance in public elementary schools is to be presented by a deputation of members of the medical profession to the President of the Board of Education on Monday next. The petition is signed by 14,718 medical men.

Dr. Robert Jones, medical superintendent of Claybury Asylum, has been awarded the silver medal of the Society of Arts, for his paper on Physical and Mental Degeneration.

It was announced at the first annual meeting of the Queen Alexandra Sanatorium at Davos, that the site had cost £6,000, and that when this was paid for, very little would remain to expend on the building. Funds are therefore much needed.

The late Mr. William Riley, of Leamington, has bequeathed a residuary interest in his estate, amounting to £1,000, to the Queen's Hospital, Birmingham, and £1,250 to the Birmingham General Hospital, and £800 to the Warneford Hospital, Leamington; in each case a bed is to be endowed and named after the testator.

Sir William Church, speaking at the annual meeting of the National Health Society, remarked that the surest way of securing the adoption of health principles was to convince the matrons of the nation of their expediency. But probably more can be done with the younger generation before they are wedded by custom to the old order of things. County Councils and other authorities can do much to encourage the teaching of health principles to the young, and if the National Health Society will help this teaching through the mouths of "babes and sucklings" we have every reason to look hopefully to the future. The mothers amongst the poor are much more likely to regard the entrance of air and the removal of dust as a fad than a blessing.

The outbreak of small-pox at Stockport at present shows no diminution.

The late Miss Mary Gregory, of Ovoca, Wicklow, has bequeathed £500 to the City of Dublin Hospital.

A concert in aid of the Russian wounded was held last Monday at the residence of Muriel, Lady Helmsley.

Dr. Roux has been appointed director, and M. Melchnikoff and M. Chamberland sub-directors of the Paris Pasteur Institute.

Since the outbreak of plague in the Transvaal, about 147 cases have been reported. Of these 120 have been amongst the coloured population.

Sir Richard Douglas Powell will deliver a lecture on the "Prevention of Consumption" before the forthcoming Sanitary Congress, which will be held in Glasgow from July 25th to 30th.

Dr. C. J. Cullingworth who has served St. Thomas's Hospital as obstetric physician for 16 years, has been appointed, on his retirement from his post, consulting obstetric physician and a governor of the hospital.

Dr. William C. Sullivan, Deputy Medical Officer H.M. Prison, Pentonville, will open a discussion on "The Criminal Responsibility of the Alcoholic" before the Society for the Study of Inebriety, in the rooms of the Medical Society of London, 11 Chandos Street, Cavendish Square, W., on Tuesday, July 12th, 1904, at 4 p.m.

The Parkes Memorial Prize of 75 guineas, open to members of the Naval, Army, and Indian Medical Services, has been awarded this year to Major R. Caldwell, R.A.M.C., for the best essay upon the "Prevention of Disease amongst Armies Engaged in the Field, with Special Reference to the Sanitary Organisation of a Field Force."

Two deputations waited upon the Police Committee of the Corporation of the City of London last week with reference to the ambulance question. Representing the Metropolitan Street Ambulance Association and the executive committee of the St. John Ambulance Association, Mr. Reginald Harrison said that they came to urge upon the Corporation of London the necessity for providing a more efficient ambulance service for the City. They were specially desirous to urge upon the committee the necessity for providing with as little delay as possible a more rapid and efficient service by the addition to the existing appliances of horse ambulances. Sir Dyce Duckworth and Sir Joseph Fayrer both laid great stress on the loss of life and unnecessary suffering due to the absence of an efficient ambulance service in London, and said they were quite ashamed of the chief city of the Empire in this respect. Sir Richard Temple, Inspector-General Ninnis, Sir William Church, and other speakers agreed with all that had been said, and strongly advocated the establishment of an efficient horse ambulance service.

